# Modelling the replacement of red and processed meat with plant-based alternatives and the estimated effect on insulin sensitivity in a cohort of Australian adults

**DOI:** 10.1017/S0007114523002659

**Published:** 2024-03-28

**Authors:** James P. Goode, Kylie J. Smith, Monique Breslin, Michelle Kilpatrick, Terence Dwyer, Alison J. Venn, Costan G. Magnussen

**Affiliations:** 1 Menzies Institute for Medical Research, University of Tasmania, Hobart, TAS 7000, Australia; 2 Heart Research Group, Murdoch Children’s Research Institute, Melbourne, VIC, Australia; 3 Nuffield Department of Women’s and Reproductive Health, University of Oxford, Oxford, UK; 4 Baker Heart and Diabetes Institute, Melbourne, VIC, Australia; 5 Centre for Population Health Research, University of Turku, Turku University Hospital, Turku, Finland; 6 Research Centre of Applied and Preventive Cardiovascular Medicine, University of Turku, Turku, Finland

**Keywords:** Cohort studies, Food, Insulin resistance, Meat, Substitution analysis

## Abstract

Dietary guidelines are increasingly promoting mostly plant-based diets, limits on red meat consumption, and plant-based sources of protein for health and environmental reasons. It is unclear how the resulting food substitutions associate with insulin resistance, a risk factor for type 2 diabetes. We modelled the replacement of red and processed meat with plant-based alternatives and the estimated effect on insulin sensitivity. We included 783 participants (55 % female) from the Childhood Determinants of Adult Health study, a population-based cohort of Australians. In adulthood, diet was assessed at three time points using FFQ: 2004–2006, 2009–2011 and 2017–2019. We calculated the average daily intake of each food group in standard serves. Insulin sensitivity was estimated from fasting glucose and insulin concentrations in 2017–2019 (aged 39–49 years) using homoeostasis model assessment. Replacing red meat with a combination of plant-based alternatives was associated with higher insulin sensitivity (*β* = 10·5 percentage points, 95 % CI (4·1, 17·4)). Adjustment for waist circumference attenuated this association by 61·7 %. Replacing red meat with either legumes, nuts/seeds or wholegrains was likewise associated with higher insulin sensitivity. Point estimates were similar but less precise when replacing processed meat with plant-based alternatives. Our modelling suggests that regularly replacing red meat, and possibly processed meat, with plant-based alternatives may associate with higher insulin sensitivity. Further, abdominal adiposity may be an important mediator in this relationship. Our findings support advice to prioritise plant-based sources of protein at the expense of red meat consumption.

## Graphical abstract

**Figure:**
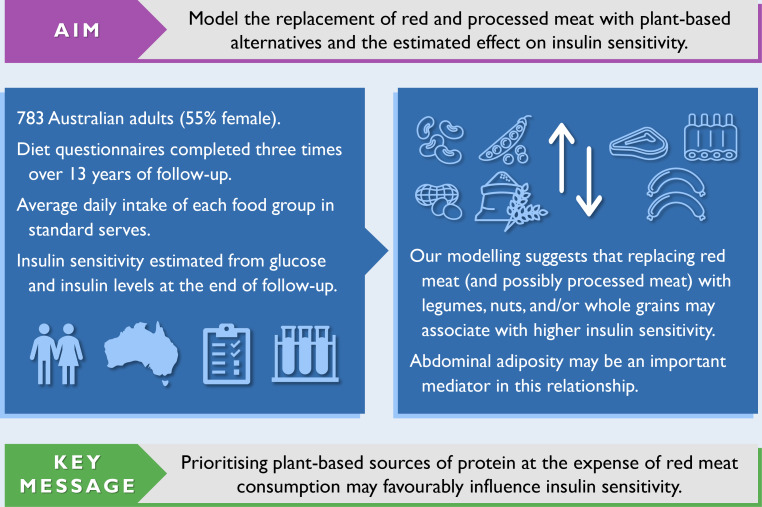

Diabetes is a chronic disease marked by elevated levels of blood glucose^(1)^. In 2021, the global prevalence of diabetes for adults aged 20–79 years was 10·5 % (537 million)^(2)^. Type 2 diabetes, which accounts for > 90 % of cases, exerts a major economic, mortality and morbidity burden worldwide^(2)^. The risk of developing type 2 diabetes can be greatly reduced by modifiable risk factors such as avoiding adiposity – a principal risk factor, not smoking, being physically active, and following established principles of healthy eating^(3,4)^.

Advice to consume plant foods such as fruit and vegetables is fundamental to most dietary guidelines^(5)^. However, explicitly encouraging a shift towards a more plant-based eating pattern for health and environmental reasons^(6)^ is a relatively recent trend. The term ‘plant-based’ generally refers to an eating pattern that prioritises plant foods (fruit, vegetables, cereals, legumes, nuts and seeds) while moderating – without necessarily excluding – animal foods (meat, eggs, dairy products and seafood)^(7)^. National dietary guidelines are starting to seriously consider health and environmental concerns when formulating recommendations^(8,9)^. Several of which explicitly promote a plant-based eating pattern and limited red meat intake (e.g. Brazil^(10)^ and Denmark^(11)^). When discussing sources of protein, dietary guidelines^(12)^ and health organisations^(13,14)^ may also preferentially encourage plant foods such as legumes, nuts, seeds and certain plant-derived products (e.g. tofu and soya ‘milk’).

In three large cohorts of American adults, higher habitual intake of red and processed meat^(15)^, including an increase over time^(16)^, was associated with elevated type 2 diabetes risk. In substitution analyses performed in the same cohorts^(15)^ and others^(17,18)^, modelling the replacement of red and processed meat with nuts or wholegrains predicted reduced type 2 diabetes risk – even after accounting for adiposity. Favourable associations were also found for legumes, but estimates were less precise and often explained by lower adiposity^(17,18)^.

Insulin resistance, where cells become less sensitive to the glucose-regulating effects of insulin^(19)^, typically marks the first stage in the pathogenesis of type 2 diabetes^(20)^. Several observational studies^(21,22)^, but not all^(23,24)^, report a positive association between the intake of protein from animal sources and insulin resistance. At the food group level, however, the longitudinal relationship between red and processed meat and insulin resistance is unclear. One large cross-sectional study found a positive association between total and processed meat intake and insulin resistance in adults, with BMI explaining much of the relationship^(25)^. The contribution of red meat, which has been implicated in other cross-sectional studies^(26,27)^, was not directly examined or modelled relative to other foods.

In nutritional epidemiology, investigations of single-food relationships often control for total energy intake to mitigate confounding^(28)^; however, in the absence of a comparison food, this can introduce a poorly defined substitution with other energy-providing foods from the background diet. To better understand the health merits of emphasising *v*. limiting a particular food choice, it is necessary to specify a comparator when modelling diet–disease relationships (e.g. what should be consumed instead of red meat?)^(29)^.

Given the growing promotion of plant-based eating patterns, coupled with advice to limit red and processed meat intake and favour plant-based sources of protein, it is important to understand how the resulting food substitutions associate with insulin resistance, a risk factor for type 2 diabetes. Compared with single-food analyses, substitution analyses specify a replacement food, making them simpler to interpret and translate into actionable recommendations. Cohort studies with repeated-measures of diet are needed to capture long-term eating patterns. Therefore, we used dietary data collected periodically over a 13-year period and statistical modelling to estimate the substitution effect of replacing red and processed meat with plant-based alternatives on insulin sensitivity in a cohort of Australian adults.

## Methods

### Study population

**Fig. 1.**
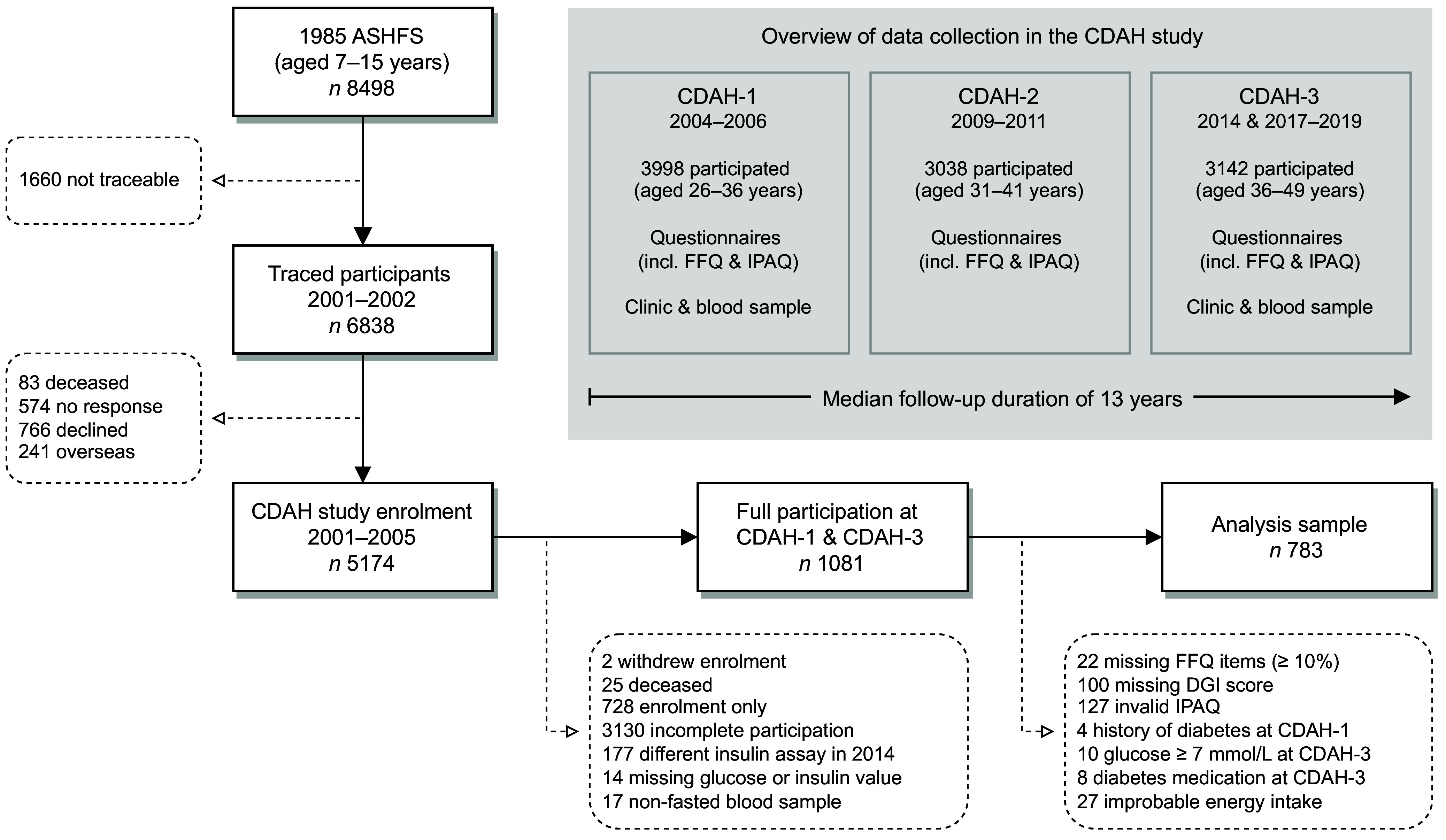
Participant flow chart and overview of data collection. ASHFS, Australian Schools Health and Fitness Survey; CDAH, Childhood Determinants of Adult Health; DGI, Dietary Guideline Index; IPAQ, International Physical Activity Questionnaire. Participants self-administered questionnaires at each time point and attended a clinic at CDAH-1 and CDAH-3 for physical measurements and blood sampling. Some participants did not complete all elements of data collection (incomplete participation). For example, a participant may have completed all questionnaires but failed to attend a clinic at CDAH-3. History of diabetes at CDAH-1 excluded cases of gestational diabetes.

The Childhood Determinants of Adult Health (CDAH) study is a mixed-sex cohort of Australians^(30)^. Participants were sourced from the 1985 Australian Schools Health and Fitness Survey, a nationally representative sample of schoolchildren aged 7–15 years^(31)^. The follow-up of successfully traced and enrolled participants has occurred at three time points in adulthood: CDAH-1 (2004–2006), CDAH-2 (2009–2011) and CDAH-3 (2014 and 2017–2019). Participants self-administered questionnaires at each time point. They also attended a clinic at CDAH-1 and CDAH-3 for physical measurements and blood sampling. Fig. 1 provides an overview of data collection. Informed consent was obtained in writing from all participants. The CDAH study and its adult follow-ups were approved by the University of Tasmania’s Human Research Ethics Committee.

### Participant selection

The analysis sample was restricted to participants who completed questionnaires at CDAH-1 and CDAH-3 and provided a fasted blood sample at CDAH-3 (*n* 1081). As the laboratory provider differed between CDAH-1 and CDAH-3, only CDAH-3 blood samples were considered to minimise potential methodological variation in insulin values. CDAH-3 blood samples from 2014 (*n* 177) were also omitted because the insulin assay differed to the one used in 2017–2019. We excluded participants with ≥ 10 % of FFQ items left blank, incomplete information on dietary habits necessary to calculate the Dietary Guideline Index score^(32)^, or an International Physical Activity Questionnaire that did not comply with accepted data cleaning and analysis procedures^(33)^. We further excluded participants with a history of type 1 or 2 diabetes at CDAH-1, a fasting plasma glucose ≥ 7 mmol/l or use of glucose-lowering medication at CDAH-3, and an improbable estimate of energy intake (i.e. > 1·5 × interquartile range above the third quartile or below the first quartile by sex). After applying exclusion criteria, 783 participants remained. Of these participants, 473 (60·4 %) also had valid dietary and physical activity data at CDAH-2. No females were pregnant at CDAH-3 blood sampling. A participant flow chart is shown in Fig. 1.

### Assessment of exposure

A qualitative FFQ with 127 food and beverage items evaluated dietary intake and habits over the previous 12 months^(34)^. This FFQ is an adapted version of the one used in the 1995 National Nutrition Survey^(35)^ and was originally developed using weighed food records in an ethnically diverse Australian population^(36)^. Participants reported their average frequency of consumption for each item using a nine-point scale, ranging from ‘never or less than once/month’ to ‘6 or more times/d’. This scale was converted to daily equivalents for analyses. No consumption was assumed for items left blank. Portion size information was not specified. The list of items was expanded to 128 at CDAH-2 and 132 at CDAH-3 to reﬂect changes in the Australian food system over time. Information was also collected on dietary habits such as the type of milk usually consumed. Our method for estimating energy intake has been described previously^(34)^. In brief, sex-specific portion sizes were assigned to items using 24-h dietary recall data from the 2011–2012 Australian National Nutrition and Physical Activity Survey, alongside energy composition values primarily from the Australian Food Composition Database (Release 1). Total energy intake was estimated by multiplying the frequency of consumption of each item by the energy content of its corresponding portion size and then summing. Alcohol intake (as ethanol) was estimated using the same approach. The intake of each item in g/d was similarly estimated using its frequency of consumption and assigned portion size.

The food groups of interest were red meat, processed meat, legumes, nuts and seeds, and wholegrains, with component foods specified in Table 1. The Australian dietary guidelines recommend a maximum red meat intake of 455 g/week, limiting the intake of processed meat, and list legumes, nuts, and seeds as alternatives to meat^(37)^. We included wholegrains because their substitution for red and processed meat has been previously associated with reduced type 2 diabetes risk^(15,16)^. The g/d intake of each food group (or component food) was scaled to the standard serve sizes used in the Australian dietary guidelines^(37)^ (Table 1). We estimated the meat component of mixed dishes using the recipe file from the 2011–2013 Australian Food and Nutrient Database^(38)^. This allowed us to gauge the average proportion of meat from a selection of common recipes.

**Table 1.** Standard serve sizes and component foods of each food group of interest

Food group	Standard serve (g)	Component foods*
Red meat	65	Mixed red meat dishes (e.g. casserole or stir-fry)†; beef or veal; mince dishes; lamb; hamburger†; pork; other offal (e.g. kidneys)
Processed meat	65	Ham or bacon; luncheon meats or salami; sausages; meat pie, sausage roll, or other savoury pastries†; liver (incl. pâté)
Legumes	75‡	Peas; green beans; soya beverages; other beans or lentils; baked beans; soyabeans or tofu
Nuts and seeds	30	Almonds, walnuts, or hazelnuts; cashews; peanut butter or other nut spreads; seeds (pumpkin, sesame, pine nuts or tahini); other nuts or seeds; peanuts; pistachio; coconuts
Wholegrains	40, bread; 30, muesli; 120, porridge	Wholemeal or mixed grain bread; muesli; cooked porridge

* Component foods are arranged in descending order from most to least frequently consumed within each food group.

† The meat component was estimated using the recipe file from the 2011–2013 Australian Food and Nutrient Database.

‡ The standard serve for soya beverages was 250 g.

### Assessment of outcome

Participants attended a clinic following an overnight fast of at least 8 h. A phlebotomist collected blood from the antecubital vein. Serum insulin was measured using an electrochemiluminescence immunoassay (Modular Analytics E170, Roche) and reported to the nearest integer. This assay was standardised to the 1^st^ International Reference Preparation for human insulin (coded 66/304) and had negligible cross-reactivity with pro-insulin. Insulin was converted from μU/ml to pmol/l using the manufacturer’s conversion factor of 6·945. Plasma glucose (mmol/l) was measured enzymatically using the hexokinase method (Siemens ADVIA 2400, Siemens Healthineers). The laboratory provider participated in the Royal College of Pathologists of Australasia Quality Assurance Programs.

We estimated insulin sensitivity from fasting glucose and insulin concentrations at CDAH-3 using homoeostasis model assessment version 2.2 (HOMA2)^(39)^. Insulin values below the acceptable range (6·5 % of total) were truncated to the lower limit of 20 pmol/l. HOMA2 is calibrated to give a ‘normal’ insulin sensitivity of 100 %; however, between population comparisons are complicated by differences in methodological factors such as choice of insulin assay^(40)^. When reporting the regression coefficient for insulin sensitivity from analyses, we refer to a change in percentage points rather than a percentage change. A higher insulin sensitivity indicates a lower degree of insulin resistance (i.e. cells are more responsive to the glucose-regulating effects of insulin).

### Assessment of other covariates

Participants self-reported information on demographics, medical history and lifestyle behaviours using a questionnaire^(30)^. The long form version of the International Physical Activity Questionnaire^(41)^ assessed the frequency, duration and intensity of all physical activities performed over the last week. Total physical activity was expressed as the metabolic equivalents (MET-h/week)^(33)^. As a measure of overall diet quality, a modified Dietary Guideline Index score was derived from FFQ responses to determine the level of compliance with the 2013 Australian dietary guidelines^(32)^. Scoring components that had substantial overlap with substituted food groups were removed (online Supplementary Table S1). Scores can range from 0 to 80. A higher score indicates better compliance with dietary recommendations. All questionnaires were supplied as hard copies to participants at CDAH-1 and CDAH-2, with a small proportion completed via computer-assisted telephone interviewing. Electronic questionnaires were introduced at CDAH-3, with 96 % of participants choosing this completion method. At clinics, technicians measured weight to 0·1 kg using portable scales (Heine) and height to 0·1 cm using a stadiometer (Invicta). BMI was calculated as weight (kg) divided by height (m) squared. Waist circumference (cm) was taken at the narrowest point between the iliac crest and the last palpable rib in the mid-axillary line.

### Statistical analyses

The average intake of each food group was calculated using available time points to reflect habitual consumption. We adopted the partition modelling approach for our substitution analysis^(42)^. This involved the use of ordinary least squares regression to estimate the effect of replacing one food group with another by including each in the model simultaneously, along with potential confounders, Dietary Guideline Index score and energy intake. Food groups not involved in a particular substitution were omitted (i.e. no mutual adjustment). Our model initially specified a 1 serve/d lower intake of red or processed meat with a concurrent 1 serve/d higher intake of legumes, nuts and seeds, or wholegrains. The difference between parameter estimates (i.e. regression coefficients and variances) and their covariance were used to estimate the ‘substitution’ effect, as detailed elsewhere^(42)^. We also halved the standard serve size of each food group listed in Table 1 to examine more modest substitutions. HOMA2 insulin sensitivity was log-transformed for analyses to improve test characteristics such as normality^(43)^. Model assumptions were checked by visual inspection of residual plots. We found no clear evidence of departures from linearity. This was also confirmed by categorising the food groups being substituted (using evenly spaced cut points) and then comparing regression coefficients at different levels of intake.

Based on putative risk factors for type 2 diabetes in Australia^(44)^, analyses were adjusted for potential confounders, including sex (male or female), age at blood draw (years), highest education at CDAH-1 (university, vocational or school), smoking status at CDAH-3 (current, former or never), physical activity (MET-h/week), parental history of diabetes (yes, no or unknown), use of hormonal contraceptives at CDAH-3 (yes or no), use of blood pressure or cholesterol-lowering medication at CDAH-3 (yes or no), energy intake (kJ/d), alcohol intake (g/d) and Dietary Guideline Index score (0–100). We adjusted for educational attainment (a dimension of socio-economic status) at CDAH-1 because it might better reflect early-life influences on lifestyle behaviours in the period leading up to outcome assessment. The average was calculated for continuous covariates evaluated at multiple time points, with physical activity, energy intake and alcohol intake further categorised into fifths using quintiles to minimise the influence of outliers.

To improve the precision of point estimates, food groups were also collapsed into two categories for analyses: (1) red and processed meat; and (2) plant-based alternatives (legumes, nuts and seeds, and wholegrains). As Australian men report a higher intake of red and processed meat than women^(45)^, we fitted interaction terms between each food group in the substitution and sex but found no substantive evidence of effect-measure modification. Thus, males and females were analysed together. Since the association between red and processed meat and type 2 diabetes risk may be partially mediated by adiposity^(15,16)^, we adjusted for waist circumference (cm) at CDAH-3 in a separate model. The difference between models was quantified as 1 − (*β*_substitution effect adjusted for adiposity_/*β*_substitution effect_). Waist circumference, a surrogate measure of abdominal adiposity, usually has a similar or stronger association with incident type 2 diabetes than BMI^(46)^.

We performed a series of sensitivity analyses. First, we adjusted for all other energy-providing food groups (in g/d) rather than energy intake and Dietary Guideline Index score. These include fruit, vegetables, tea and coffee, fruit and vegetable juices, salad dressings, refined grains, milk, yogurt, cheese, eggs, seafood, poultry, sugar-sweetened beverages, artificially sweetened beverages, takeaway foods, and mixed discretionary foods (online Supplementary Table S2). This ‘all-components model’ was developed using a causal inference framework by Tomova *et al*. and has been proposed as a more robust modelling strategy^(47,48)^, the details and implications of which are still being discussed in relation to current practices^(49–51)^. Second, we excluded participants with a self-reported history of CVD, hypertension, hypercholesterolaemia or polycystic ovary syndrome at CDAH-1 because these health conditions may have prompted diet and lifestyle changes, possibly resulting in reverse causation bias. Third, we excluded participants who reported < 0·3 serve/d of red meat or plant-based alternatives to assess the influence of infrequent and non-consumers. Lastly, we performed an inverse probability weighting procedure to mitigate any possible bias due to differential loss to follow-up, as described previously^(52)^. We identified participant characteristics from the 1985 Australian Schools Health and Fitness Survey that were associated with loss to follow-up (online Supplementary Table S3). Multiple imputation accounted for missing values among participant characteristics using fully conditional specification models to impute fifty datasets (under the missing at random assumption). For each dataset, a model for the probability of being included in the analysis sample was fit and each participant was then assigned a weight corresponding to the inverse of their probability of being included. Our analysis was repeated using the weights derived from each imputed dataset. Regression coefficients were averaged across analyses to provide a final point estimate. The accompanying standard error was estimated using Rubin’s rules. The characteristics of participants from the Australian Schools Health and Fitness Survey, the CDAH study and our analysis sample are compared in Supplementary Table S3.

Statistical analyses were performed using Stata 17 (StataCorp.). The command xtile ranked participants into categories using quantiles as cut points^(53)^. We present two-tailed *P*-values and interpret them as a continuous measure of compatibility between our data and a test hypothesis of no association^(54)^.

## Results

The median follow-up duration between questionnaire completion at CDAH-1 and CDAH-3 was 13 years (range, 11–14·5 years). Participant characteristics of the overall analysis sample and by joint stratification of exposure intake categories are shown in Table 2. Dietary characteristics are likewise shown in Table 3. Participants with a higher intake of plant-based alternatives (and a lower intake of red and processed meat) were more often female, university-educated, a non-smoker and not taking blood pressure or cholesterol-lowering medication. They also tended to have had a much lower BMI and waist circumference, and higher estimates of insulin sensitivity. Regarding habitual diet across the follow-up period, participants with a higher intake of plant-based alternatives (and a lower intake of red and processed meat) also had a lower intake of energy and alcohol, and better compliance with the Australian dietary guidelines. When stratified by sex, however, energy intake was similar across categories (data not shown). Other notable differences in habitual diet include higher intakes of fruit, vegetables, tea and coffee, and yogurt, and lower intakes of refined grains, milk, poultry, and sugar-sweetened beverages (online Supplementary Table S4).

**Table 2.** Characteristics of the overall analysis sample (*n* 783) and by joint stratification of exposure intake categories at CDAH-3*

Characteristic	Overall		Low-high		Middle		High-low	
	*n*	%	*n*	%	*n*	%	*n*	%
Sex (female)	428	54·7	28	27·7	61	64·9	79	73·8
	Mean	sd	Mean	sd	Mean	sd	Mean	sd
Age (years)	44·5	2·6	44·4	2·4	44·5	2·7	44·3	2·6
	*n*	%	*n*	%	*n*	%	*n*	%
Highest education at CDAH-1								
University	414	52·9	31	30·7	49	52·1	73	68·2
Vocational	213	27·2	44	43·6	28	29·8	22	20·6
School	156	19·9	26	25·7	17	18·1	12	11·2
Smoking status								
Never	516	65·9	63	62·4	59	62·8	68	63·6
Former	207	26·4	28	27·7	29	30·9	35	32·7
Current	60	7·7	10	9·9	6	6·4	4	3·7
	Median	IQR	Median	IQR	Median	IQR	Median	IQR
Physical activity (MET-h/week)†	46	30–67	53	37–74	45	31–61	48	30–68
	Mean	sd	Mean	sd	Mean	sd	Mean	sd
BMI (kg/m^2^)	27·1	5·0	29·0	5·3	27·2	4·6	24·9	4·0
Waist circumference (cm)§	87·9	12·8	94·4	12·4	87·2	11·6	82·4	10·7
	*n*	%	*n*	%	*n*	%	*n*	%
Parental history of diabetes	60	7·7	8	7·9	9	9·6	4	3·7
Hormonal contraceptive use	114	14·6	11	10·9	19	20·2	13	12·2
Current medication use‡	42	5·4	11	10·9	4	4·3	2	1·9
	Mean	sd	Mean	sd	Mean	sd	Mean	sd
Plasma glucose (mmol/l)	4·7	0·4	4·8	0·4	4·8	0·5	4·7	0·4
	Median	IQR	Median	IQR	Median	IQR	Median	IQR
Serum insulin (pmol/l)	42	28–56	49	31–69	42	28–56	28	21–49
HOMA2 insulin sensitivity (%)	122	84–172	96	67–154	116	82–172	166	101–229

CDAH, childhood determinants of adult health; HOMA2, homoeostasis model assessment version 2·2; IQR, interquartile range; MET, metabolic equivalents.

* Participants were jointly stratified by opposing thirds of intake of: (1) plant-based alternatives; and (2) red and processed meat: lowest third of alternatives and highest third of meat (low-high, *n* 101), middle third of both alternatives and meat (middle, *n* 94), and highest third of alternatives and lowest third of meat (high-low, *n* 107). Participant data for the other six joint categories are not shown.

† Average across available time points (CDAH-1 and CDAH-3, and if available, CDAH-2). Overall, 515 participants (65·8 %) also had physical activity data at CDAH-2.

‡ Current use of either blood pressure or cholesterol-lowering medication.

§ *n* 781 due to missing data.

**Table 3.** Dietary characteristics of the overall analysis sample (*n* 783) and by joint stratification of exposure intake categories*

Characteristic†	Overall		Low-high		Middle		High-low	
	Mean	sd	Mean	sd	Mean	sd	Mean	sd
Total energy intake (MJ/d)	9·6	2·6	10·5	2·3	9·2	1·8	9·2	2·0
Alcohol intake (g/d)	17·2	18·8	21·8	23·4	18·4	18·4	14·7	13·5
	*n*	%	*n*	%	*n*	%	*n*	%
Abstainers	59	7·5	6	5·9	6	6·4	8	7·5
	Mean	sd	Mean	sd	Mean	sd	Mean	sd
Dietary guideline index score	41·5	8·4	34·6	7·0	41·9	7·4	47·4	7·3
Red and processed meat‡	1·8	0·9	2·8	0·8	1·7	0·2	0·8	0·4
Red meat	1·3	0·7	2·0	0·7	1·3	0·3	0·6	0·4
Processed meat	0·4	0·3	0·7	0·4	0·4	0·3	0·2	0·1
Plant-based alternatives‡	2·6	1·6	1·0	0·4	2·3	0·4	4·5	1·2
Legumes	0·6	0·5	0·3	0·2	0·5	0·3	1·1	0·7
Nuts and seeds	0·5	0·5	0·2	0·2	0·4	0·3	1·0	0·7
Wholegrains	1·5	1·1	0·5	0·3	1·4	0·5	2·4	1·0

* Participants were jointly stratified by opposing thirds of intake of: (1) plant-based alternatives; and (2) red and processed meat: lowest third of alternatives and highest third of meat (low-high, *n* 101), middle third of both alternatives and meat (middle, *n* 94), and highest third of alternatives and lowest third of meat (high-low, *n* 107). Participant data for the other six joint categories are not shown.

† Average across available time points (CDAH-1 and CDAH-3, and if available, CDAH-2). Almost two-thirds completed a FFQ at CDAH-2: 496 had a Dietary Guideline Index score and 531 had an estimate for remaining dietary variables.

‡ The g/d intake was scaled to the standard serve sizes used in the Australian dietary guidelines.

There was a negative correlation between the intake of plant-based alternatives and red and processed meat (*r* = –0·17, *P* < 0·001). There was also a positive correlation between the intake of red meat and processed meat (*r* = 0·42, *P* < 0·001). Overall, the intake of plant-based alternatives (legumes, nuts and seeds, and wholegrains) and red and processed meat remained relatively stable in the analysis of sample across the three time points (online Supplementary Table S5). This is excepting nuts and seeds, which increased from 0·3 serve/d at CDAH-1 to 0·7 serve/d at CDAH-3.

The results of our confounder-adjusted substitution analyses are presented in Table 4, alongside unadjusted results for comparison purposes. Replacing red meat with legumes, nuts and seeds, or wholegrains was associated with higher insulin sensitivity. The substitution involving nuts and seeds had the largest point estimate. Collapsing individual food groups into a combined plant-based alternatives category produced a similar result, but with a narrower CI. Replacing processed meat with legumes, nuts and seeds, or wholegrains was also associated with higher insulin sensitivity; however, while point estimates were similar, CI were much wider. Further, collapsing individual food groups into a combined plant-based alternatives category had little impact on the width of CI. Combining red and processed meat into the same category yielded similar results to the substitution involving only red meat, but with slightly smaller point estimates and narrower CI. When back-transformed from the logarithmic scale, replacing 1 serve/d of red meat with 1 serve/d of plant-based alternatives was associated with a higher estimate of insulin sensitivity (*β* = 10·5 %, 95 % CI (4·1, 17·4)). The associations with higher insulin sensitivity persisted after halving the modelled replacement of 1 serve/d to 0·5 serve/d for each food group (online Supplementary Table S6). Results were virtually unchanged when adjusting for the original Dietary Guideline Index score (as opposed to a modified score with components removed that had substantial overlap with substituted food groups).

**Table 4.** Modelled replacement of red and processed meat with plant-based alternatives and the estimated effect on log-HOMA2 insulin sensitivity (*n* 783)*

	Unadjusted model			Adjusted model†		
	*β*	95 % CI	*P*	*β*	95 % CI	*P*
Replacing red meat with…						
Plant-based alternatives	0·13	0·07, 0·18	<0·001	0·10	0·04, 0·16	0·001
Legumes	0·18	0·09, 0·27	<0·001	0·12	0·03, 0·21	0·010
Nuts and seeds	0·21	0·12, 0·29	<0·001	0·15	0·06, 0·24	0·001
Wholegrains	0·15	0·09, 0·21	<0·001	0·11	0·05, 0·18	0·001
Replacing processed meat with…						
Plant-based alternatives	0·16	0·05, 0·27	0·006	0·09	–0·06, 0·23	0·24
Legumes	0·22	0·09, 0·35	0·001	0·13	–0·03, 0·29	0·10
Nuts and seeds	0·24	0·12, 0·37	<0·001	0·15	–0·003, 0·30	0·054
Wholegrains	0·20	0·08, 0·32	0·001	0·12	–0·03, 0·26	0·12
Replacing red and processed meat with…						
Plant-based alternatives	0·11	0·07, 0·16	<0·001	0·09	0·04, 0·14	0·001
Legumes	0·16	0·08, 0·25	<0·001	0·10	0·02, 0·19	0·016
Nuts and seeds	0·19	0·11, 0·27	<0·001	0·14	0·05, 0·22	0·001
Wholegrains	0·14	0·09, 0·19	<0·001	0·10	0·04, 0·16	<0·001

HOMA2, homoeostasis model assessment version 2.2.

* The change in log-transformed HOMA2 insulin sensitivity (percentage points) when simulating a 1 serve/d lower intake of red and processed meat with a concurrent 1 serve/d higher intake of plant-based alternatives (legumes, nuts and seeds, and wholegrains).

† Adjusted for sex, age at blood draw, highest education, smoking status, physical activity, parental history of diabetes, use of hormonal contraceptive, use of blood pressure or cholesterol-lowering medication, energy intake, alcohol intake and Dietary Guideline Index score.

The results of our sensitivity analyses are presented in Supplementary Table S7. In these analyses, we specified a 1 serve/d lower intake of red meat with a concurrent 1 serve/d higher intake of plant-based alternatives. Further adjustment for waist circumference attenuated the point estimate by 61·7 %, eliminating most of the prior association with higher insulin sensitivity. Use of the all-components model moderately attenuated the point estimate, but the association with higher insulin sensitivity remained. In this case, further adjustment for waist circumference attenuated the point estimate by 41 %. Of note, when comparing our original model to the all-components model, the inclusion of waist circumference resulted in nearly identical parameter estimates. The exclusion of participants with a health condition at CDAH-1, or those who typically consumed < 0·3 serve/d of red meat or plant-based alternatives, did not appreciably change our results. Similarly, the association with higher insulin sensitivity remained after applying our inverse probability weighting procedure, but doing so slightly attenuated the point estimate and widened the CI.

## Discussion

In our substitution analyses, modelling the replacement of red meat with legumes, nuts and seeds, or wholegrains (or a combination thereof) was associated with higher insulin sensitivity in Australian adults. Similar but less precise estimates were found for processed meat. When replacing red meat with a combination of plant-based alternatives, waist circumference strongly attenuated the association with higher insulin sensitivity.

We advance prior evidence from cross-sectional studies associating red and processed meat intake with surrogate markers of insulin resistance^(25–27)^ by modelling average intakes over a 13-year period and suitable replacement foods of public health interest. Our findings complement a substitution analysis by Ley *et al.*, where exchanging red and processed meat for nuts or legumes was linked with lower levels of fasting insulin^(55)^. We estimated our substitution effects by comparing average intakes rather than active changes. However, our results are consistent with substitution analyses that modelled longitudinal changes in red meat intake and subsequent risk of type 2 diabetes^(56,57)^. While a confirmatory randomised controlled trial would be ideal, numerous logistical challenges hinder the completion of multi-year trials^(58)^. Instead, we present data from a long-term cohort study – the next best line of evidence.

In Western populations, red and processed meat intake is consistently associated with type 2 diabetes risk^(59,60)^, with a stronger effect for processed meat. However, our substitution effects for processed meat were similar (and much less precise) relative to red meat. The reason for this similarity and lack of precision is unclear, but possible explanations include a narrow distribution of processed meat intake, underreporting, portion size errors and insufficient statistical power. Nonetheless, our results agree with several substitution analyses that examined red and processed meat and type 2 diabetes risk^(15,17,18)^.

We scaled substituted foods using standard serves to aid interpretation, but these may not reflect typical portion sizes^(61)^. In Australia (2011–2012), the median adult portion size of red meat was 75 g at lunch and 104 g at dinner, and about 140–160 g when choosing steak^(62)^. We initially modelled a 1 serve/d (65 g/d) lower intake of red meat but also found a favourable association with insulin sensitivity when substituting 0·5 serve/d. Thus, when replaced with specific plant foods, even modest reductions in red meat may contribute to lower type 2 diabetes risk, as found recently^(63)^. For context, some authorities advise < 350 g/week of red meat for health^(13)^ and environmental reasons^(64,65)^, but in Australia, 59 % of men and 33 % of women may regularly exceed 455 g/week^(45)^. Lowering red meat intake could be achieved by reducing portion sizes, designating certain meals as meat-free or partial replacement with other foods. Certain food substitutions are applicable to a single-meal setting (e.g. altering the proportion of red meat and legumes in a recipe), while others may require shifts in food choices throughout the day (e.g. choosing a meat-free lunch and to snack on nuts). A plant-based meat substitute could also be a convenient one-to-one replacement for red meat. As a compromise (or an intermediary step), a more palatable change could be to replace red meat with fish or poultry, which may^(56)^ (or may not^(57)^) lower type 2 diabetes risk.

In terms of public health messaging, our modelling of food groups is conducive to the formulation of actionable recommendations. This contrasts with the less practical – though more aetiologically relevant – units such as the amount of protein or energy from a particular source. However, due to holding total energy intake constant and the divergent energy content among substituted foods, our models do not account for the resulting – albeit modest – residual difference in energy intake^(66)^. Based on the food modelling system for Australia, the approximate energy content per standard serve is 550 kJ for lean red meat, 350 kJ for legumes, 750 kJ for nuts and seeds, and 450 kJ for wholegrains and higher-fibre cereals^(67)^. When substituting foods in practice, a further consideration is the provision of essential nutrients. For example, when substituting red meat with legumes, the background diet may need to ensure other reliable sources of vitamin B_12_ (such as poultry, dairy products, seafood, fortified foods or a dietary supplement)^(68,69)^.

Within a main meal context, exchanging meat for plant-based alternatives may also influence accompanying food choices, resulting in changes to the underlying eating pattern^(70)^ that could differentially – and independently – associate with health outcomes. Whether to adjust for other foods in a substitution analysis will depend on the research question, as doing so may artificially restrict the underlying eating pattern^(66)^. This would undermine the public health relevance but offer insight from an aetiological perspective if other foods are considered confounders rather than co-occurring factors with their own additional benefits. Adjusting for all other food groups (i.e. the all-components model) moderately attenuated our point estimate, but the association with higher insulin sensitivity remained when replacing red meat with plant-based alternatives. This suggests both public health and aetiological relevance. In addition to direct effects, our substituted foods may be indicators of an underlying eating pattern that also associates favourably with insulin sensitivity.

Insulin resistance is an early defect in the pathogenesis of type 2 diabetes that progressively worsens over time, particularly in the years prior to diagnosis^(71)^. Homoeostasis model assessment is widely used in epidemiological studies as a convenient, surrogate measure of insulin sensitivity^(39)^. It has been validated against direct measurement techniques such as the hyperinsulinemic–euglycemic clamp^(72)^. Since estimates are derived from fasting glucose and insulin concentrations, homoeostasis model assessment principally describes hepatic insulin sensitivity^(73)^. A higher estimate of insulin sensitivity generally predicts lower type 2 diabetes risk, but other risk factors such as obesity are still important^(74–76)^. In our population, we infer that habitually replacing red meat with plant-based alternatives may also translate into lower type 2 diabetes risk, as found previously^(15)^.

The dominant role of excess body fat (principally in the liver and pancreas) in the development of insulin resistance and *β*-cell dysfunction is well established^(77)^. When replacing red meat with plant-based alternatives, waist circumference attenuated the association with higher insulin sensitivity by about 40–60 %. Thus, abdominal adiposity may be an important mediator in this relationship. This is in line with a formal mediation analysis by Mazidi *et al*., where nearly half of the association between red meat intake and insulin sensitivity was explained by waist circumference^(78)^. In general, meat consumption is associated with weight gain in European populations^(79)^, but whether this relationship is causal, and the possible extent of residual confounding, is unclear.

Aside from adiposity-related mechanisms, several components of red meat may contribute to the development of insulin resistance and type 2 diabetes, as reviewed previously^(80–82)^. Examples include branched-chain amino acids, haem iron, advanced glycation end products formed by the Maillard (or ‘browning’) reaction, and phosphatidylcholine and l-carnitine – and their eventual conversion to trimethylamine N-oxide. Potential mechanisms include oxidative stress, elevations in inflammatory markers, disruption to insulin signalling pathways and damage to insulin-producing *β*-cells. In contrast, intakes of legumes, nuts and seeds, and wholegrains generally improve markers of glucose homoeostasis (fasting glucose, glycated haemoglobin and insulin sensitivity)^(83)^. Likely beneficial components of these foods include antioxidants, phytochemicals, unsaturated fatty acids and fibre^(84)^. A higher intake of cereal fibre appears particularly beneficial and, mechanistically, may enhance satiety and weight maintenance, lower inflammatory markers, and augment gut microbiota composition and the production of SCFA via fermentation^(85,86)^.

Assuming we sufficiently predicted loss to follow-up, our inverse probability weighting procedure did not reveal substantive selection bias; however, we may have slightly overestimated our point estimate due to the characteristics of our analysis sample relative to the source population. Excluding participants with a health condition that may have prompted lifestyle changes did not change our results, helping to mitigate reverse causation bias. The limited number of infrequent and non-consumers of red meat (or plant-based alternatives) had little impact on our estimates. Our findings may not be generalisable to populations with different levels of food group intake, underlying eating patterns and socio-economic status. The composition of food groups and culinary practices may also vary. Taking red meat as an example, certain cooking methods (e.g. roasting or barbequing) and a preference for higher doneness may increase type 2 diabetes risk independent of total meat consumption^(87)^.

Despite adjusting for potential confounders, unmeasured confounding remains a concern in observational studies. Residual confounding is likewise a concern for imperfectly measured covariates (e.g. physical activity and energy intake). In European countries^(88)^ and the USA^(89)^, higher meat intake is generally accompanied by higher rates of smoking, obesity and lower educational attainment, among other differences. We observed a similar profile of characteristics among participants with higher intakes of red and processed meat (and lower intakes of plant-based alternatives), including notable differences in diet quality. The unfavourable association between red meat and insulin resistance may be partly explained by lifestyle and dietary factors that cluster at different levels of red meat intake. Therefore, the results of the present study should be interpreted with caution due to the possibility of unmeasured and residual confounding.

FFQ data are subject to measurement error. We modelled average intakes to help reduce within-person variation and account for changes in intake over time. Compared with biomarker-calibrated red meat intake, the association between unadjusted intake and type 2 diabetes risk is substantially attenuated^(90)^. Therefore, we may have underestimated our estimated substitution effects due to measurement error. Further, changes in multiple dietary components, rather than a select few, may also result in larger effect sizes. Due to a lack of detailed information, some wholegrain (or high-fibre) foods may have been misclassified as refined grains, possibly resulting in an underestimation of wholegrain intake from sources such as rice, pasta and ready-to-eat breakfast cereals.

In conclusion, our modelling found that replacing red meat, and possibly processed meat, with legumes, nuts and seeds, or wholegrains (or a combination thereof) predicted higher insulin sensitivity in Australian adults, implying a lower risk of type 2 diabetes. These findings are timely given the increasing promotion of mostly plant-based diets, limits on red meat consumption, and plant-based sources of protein for health and environmental reasons. The rapid emergence and commercialisation of plant-based meat substitutes, as opposed to farmed meat, may present further avenues of investigation for public health researchers.

## Supporting information

Goode et al. supplementary material Goode et al. supplementary material

## Supplementary material

For supplementary material/s referred to in this article, please visit https://doi.org/10.1017/S0007114523002659
