# Supplementary material for: Modelling the replacement of red and processed meat with plant-based alternatives and the estimated effect on insulin sensitivity in a cohort of Australian adults
Source: Br J Nutr. 2023 Nov 20;131(6):1084–94. doi: 10.1017/S0007114523002659 (PMC10876457; doi:10.1017/S0007114523002659)
Supplement: Goode et al. supplementary material [file S0007114523002659sup001.docx]

**Supplemental Table S1.** Modified Dietary Guideline Index scoring matrix based on the 2013 Australian dietary guidelines for adults aged 19–50 years*

| Dietary Guideline | # | Indicator and description | Max score | Criteria for maximum score | | Criteria for minimum score |
| --- | --- | --- | --- | --- | --- | --- |
|  |  |  |  | Men | Women |  |
| Enjoy a variety of nutritious foods | 1 | Intake of foods from each of the five core food groups | 10 | 1 serve from each core food group  (2 points each) | | 0 serves from any of the core food groups |
| Vegetables | 2 | Serves of vegetables per day | 10 | ≥ 6 | ≥ 5 | 0 |
| Fruit | 3 | Serves of fruit per day (max. 125 ml 100% fruit juice, 1 serve of dried fruit) | 10 | ≥ 2 | ≥ 2 | 0 |
| Grain foods, mostly whole grain and/or high-fibre varieties | 4a | Serves of bread and grains (cereals) per day | 5 | ≥ 6 | ≥ 6 | 0 |
|  | 4b | Proportion of whole grain bread to total bread | 5 | 100% | 100% | 0% |
| Lean meats and poultry, fish, eggs, tofu, nuts/seeds, and legumes/beans | 5a | Serves of meat or alternatives per day (excluding processed meat) | 5 | ≥ 3 | ≥ 2·5 | 0 |
|  | 5b | Proportion of lean meats/alternatives to total meat/alternatives | 5 | 100% | 100% | 0% |
| Dairy and/or alternatives, mostly reduced fat | 6a | Serves per day of total dairy or alternatives | 5 | ≥ 2·5 | ≥ 2·5 | 0 |
|  | 6b | Type of milk usually consumed | 5 | Skimmed or reduced-fat milk or alternatives | | Whole milk |
| Drink plenty of water | 7a | Serves of fluids per day, excluding alcohol† | 5 | ≥ 10 | ≥ 8 | 0 |
|  | 7b | Proportion of water to total fluid intake per day, excluding alcohol‡ | 5 | ≥ 50% | ≥ 50% | 0% |
| Limit intake of foods containing saturated fat, added salt/sugar, and alcohol | 8 | Serves per day of alcohol and foods high in saturated fat or added salt/sugars | 20 | ≤ 1·5 | ≤ 1·25 | > 3 for men  > 2.5 for women |
| Replace sources of saturated fat with unsaturated fat | 9a | How regularly is fat trimmed from meat | 5 | Usually done | Usually done | Never or rarely |
|  | 9b | Type of spread usually used on bread, etc. | 5 | Spreads low in saturated fat | | Spreads high in saturated fat |

*As per Wilson et al.,^(1)^ Dietary Guideline Index scores were derived from food frequency questionnaire data, assuming 1 standard serve was consumed at each eating occasion. Scoring components that had substantial overlap with substituted food groups were removed (4a, 4b, 5a, and 5b). Thus, modified scores can range from 0–80, with a higher score reflecting better compliance with dietary guidelines. Proportional scores were awarded for partially met criteria. For example, 2 serves of fruit would score 10 points (maximum), 1 serve would score 5 points, and no intake would score 0 points (minimum).

†Fluid intake cut-offs are based on the 2006 Nutrient Reference Values for Australia and New Zealand.^(2)^

‡The cut-offs for the proportion of water to total fluids was informed by recommendations from the United States Beverage Guideline Panel.^(3)^

**Supplemental Table S2.** Component foods of other energy-providing food groups

| Food group | Component foods* |
| --- | --- |
| Fruit | Apple or pear; banana; avocado; grapes or berries; orange, mandarin, or grapefruit; fruits (dried, frozen, or canned); melon; peach, nectarine, plum, or apricot; fresh fruit salad; pineapple; mango or papaya; other fruit not listed; kiwifruit |
| Vegetables | Carrots; mixed salad (as a side salad or in a sandwich); stir-fried or mixed vegetables; onion or leek; broccoli; potatoes; tomatoes; capsicum; cucumber; mushrooms; lettuce; cauliflower; sweetcorn; zucchini, eggplant, or squash; pumpkin; silverbeet, spinach, or Asian greens; sweet potato; Brussels sprout, cabbage, or coleslaw; beetroot; celery; vegetable casserole |
| Tea and coffee | Coffee; green or black tea; herbal tea; decaffeinated coffee |
| Fruit and vegetable juices | Fruit juice (100%); vegetable or tomato juice |
| Salad dressings | Oil and vinegar dressing; mayonnaise or other creamy dressings |
| Refined grains | White bread; breakfast cereal; pasta or noodles; dry or savoury biscuits, crispbread, or crackers; rice (white or brown); flat bread; English muffin, bagel, or crumpet |
| Milk | Milk; flavoured milk drink |
| Yoghurt | Yoghurt (plain or flavoured) |
| Cheese | Cheddar and other cheeses; cream cheese; cottage or ricotta cheese |
| Eggs | Eggs |
| Seafood | Canned fish; fresh fish; frozen fish; prawns; calamari or squid; other seafood; mussels or oysters; crustaceans (lobster or crayfish) |
| Poultry | Mixed poultry dishes; chicken, turkey, or duck |
| Sugar-sweetened beverages | Soft drinks; fruit juice drink or fruit cordial; cordial; energy drinks |
| Artificially sweetened beverages | Low-joule soft drink; low-joule cordial |
| Takeaway foods | Hot chips, roast potatoes, or potato wedges; pizza; fried chicken; fried fish |
| Mixed discretionary foods | Sugar added to coffee or tea; chocolate; plain or sweet biscuits; jam, marmalade, syrup, or honey; cakes, sweet muffins, scones, or pikelets; potato chips or corn chips; other confectionaries; cream or chocolate biscuits; ice-cream; cream or sour cream; other pudding or desserts; creamy dips and spreads; sweet pies or pastries |

*Component foods are arranged in descending order from most to least frequently consumed within each food group. Each component corresponds to a food frequency questionnaire item.

**Supplemental Table S3.** Characteristics of participants from the 1985 Australian Schools Health and Fitness Survey (*n* 8498) and those restricted to the CDAH study (*n* 5172) and our analysis sample (*n* 783)*

|  | Australian Schools Health and Fitness Survey | | |  | CDAH study | |  | Analysis sample | |
| --- | --- | --- | --- | --- | --- | --- | --- | --- | --- |
| Characteristic | *n* | % | *N* |  | *n* | % |  | *n* | % |
| Sex |  |  | 8498 |  |  |  |  |  |  |
| Male | 4307 | 50·7 |  |  | 2439 | 47·2 |  | 355 | 45·3 |
| Female | 4191 | 49·3 |  |  | 2733 | 52·8 |  | 428 | 54·7 |
| Age (year) |  |  | 8498 |  |  |  |  |  |  |
| Mean (SD) | 10·9 | (2·5) |  |  | 11·0 | (2·5) |  | 11·2 | (2·5) |
| School classification |  |  | 8498 |  |  |  |  |  |  |
| Government | 6375 | 75·0 |  |  | 3785 | 73·2 |  | 558 | 71·3 |
| Catholic | 1673 | 19·7 |  |  | 1077 | 20·8 |  | 168 | 21·5 |
| Independent | 450 | 5·3 |  |  | 310 | 6·0 |  | 57 | 7·3 |
| Teacher-rated scholastic ability |  |  | 7960 |  |  |  |  |  |  |
| Excellent | 743 | 9·3 |  |  | 500 | 10·3 |  | 108 | 14·6 |
| Above average | 2211 | 27·8 |  |  | 1486 | 30·5 |  | 283 | 38·4 |
| Average | 3279 | 41·2 |  |  | 2004 | 41·2 |  | 265 | 35·9 |
| Below average | 1335 | 16·8 |  |  | 702 | 14·4 |  | 73 | 9·9 |
| Poor | 392 | 4·9 |  |  | 174 | 3·6 |  | 9 | 1·2 |
| Language spoken at home |  |  | 6395 |  |  |  |  |  |  |
| English | 5575 | 87·2 |  |  | 3529 | 88·9 |  | 585 | 92·4 |
| European | 608 | 9·5 |  |  | 344 | 8·7 |  | 37 | 5·9 |
| Other (non-specified) | 212 | 3·3 |  |  | 96 | 2·4 |  | 11 | 1·7 |
| Parental smoking status |  |  | 6372 |  |  |  |  |  |  |
| Non-smokers | 3217 | 50·5 |  |  | 2125 | 53·7 |  | 407 | 64·6 |
| Smokers | 1046 | 16·4 |  |  | 580 | 14·7 |  | 65 | 10·3 |
| Mother smokes | 775 | 12·2 |  |  | 439 | 11·1 |  | 44 | 7·0 |
| Father smokes | 1334 | 21·0 |  |  | 813 | 20·6 |  | 114 | 18·1 |
| Relative socio-economic disadvantage† |  |  | 6298 |  |  |  |  |  |  |
| Quarter 1 (most disadvantaged) | 582 | 9·2 |  |  | 307 | 7·9 |  | 33 | 5·3 |
| Quarter 2 | 2427 | 38·5 |  |  | 1554 | 39·9 |  | 218 | 35·2 |
| Quarter 3 | 1799 | 28·6 |  |  | 1076 | 27·6 |  | 193 | 31·2 |
| Quarter 4 (least disadvantaged) | 1490 | 23·7 |  |  | 962 | 24·7 |  | 175 | 28·3 |
| Body mass index (kg/m^2^) categories‡ |  |  | 8490 |  |  |  |  |  |  |
| Normal | 7490 | 88·2 |  |  | 4622 | 89·4 |  | 734 | 94·0 |
| Overweight | 862 | 10·2 |  |  | 476 | 9·2 |  | 44 | 5·6 |
| Obese | 138 | 1·6 |  |  | 72 | 1·4 |  | 3 | 0·4 |

CDAH, Childhood Determinants of Adult Health; SD, standard deviation.

*Presented data were collected in 1985 as part of the Australian Schools Health and Fitness Survey.

†The Index of Relative Socio-Economic Disadvantage ranks geographical areas in Australia based on the prevalence of certain economic and social conditions.^(4)^ Index scores were assigned using postcode of residence, and then categorised into fourths of the distribution.

‡Cut-off points for categories were age- and sex-specific.^(5)^

**Supplemental Table S4.** Intake of other food groups in our analysis sample (*n* 783) and by joint stratification of exposure intake categories*

|  | Overall | |  | Low-High | |  | Middle | |  | High-Low | |
| --- | --- | --- | --- | --- | --- | --- | --- | --- | --- | --- | --- |
| Food group (g/day)† | p50 | (IQR) |  | p50 | (IQR) |  | p50 | (IQR) |  | p50 | (IQR) |
| Fruit | 225 | 133, 347 |  | 133 | 78, 213 |  | 252 | 160, 348 |  | 334 | 223, 448 |
| Vegetables | 315 | 244, 403 |  | 284 | 227, 365 |  | 305 | 246, 385 |  | 362 | 294, 444 |
| Tea and coffee | 534 | 275, 771 |  | 363 | 118, 629 |  | 567 | 359, 773 |  | 579 | 344, 766 |
| Fruit and vegetable juices | 67 | 22, 142 |  | 58 | 21, 142 |  | 68 | 22, 158 |  | 72 | 29, 142 |
| Salad dressings | 5 | 2, 9 |  | 4 | 2, 8 |  | 4 | 2, 7 |  | 5 | 3, 10 |
| Refined grains | 153 | 110, 205 |  | 166 | 124, 217 |  | 150 | 111, 205 |  | 136 | 106, 185 |
| Milk | 191 | 116, 288 |  | 221 | 119, 330 |  | 189 | 122, 280 |  | 155 | 87, 214 |
| Yoghurt | 24 | 6, 54 |  | 7 | 0, 18 |  | 30 | 10, 54 |  | 37 | 9, 66 |
| Cheese | 12 | 7, 17 |  | 12 | 9, 17 |  | 11 | 8, 15 |  | 13 | 8, 18 |
| Eggs | 12 | 7, 21 |  | 14 | 6, 20 |  | 12 | 7, 20 |  | 14 | 7, 22 |
| Seafood | 25 | 13, 38 |  | 20 | 10, 30 |  | 22 | 14, 36 |  | 27 | 17, 48 |
| Poultry | 66 | 41, 100 |  | 71 | 48, 105 |  | 63 | 46, 98 |  | 44 | 24, 73 |
| Sugar-sweetened beverages | 93 | 27, 273 |  | 333 | 147, 534 |  | 76 | 32, 147 |  | 25 | 7, 90 |
| Artificially sweetened beverages | 8 | 0, 79 |  | 12 | 0, 81 |  | 9 | 0, 98 |  | 0 | 0, 18 |
| Takeaway foods | 34 | 22, 48 |  | 48 | 38, 67 |  | 30 | 20, 42 |  | 25 | 14, 37 |
| Mixed discretionary foods | 66 | 43, 97 |  | 77 | 54, 116 |  | 75 | 42, 96 |  | 59 | 35, 83 |

SD, standard deviation; IQR, interquartile range; p50, median (50^th^ percentile).

*Participants were jointly stratified by opposing thirds of intake of (1) plant-based alternatives and (2) red and processed meat: lowest third of alternatives and highest third of meat (Low-High, *n* 101), middle third of both alternatives and meat (Middle, *n* 94), and highest third of alternatives and lowest third of meat (High-Low, *n* 107). Participant data for the other six joint categories are not shown.

†Average across available time points (CDAH-1 and CDAH-3, and if available, CDAH-2). Overall, 531 participants (63·3%) also had food group intake data at CDAH-2.

**Supplemental Table S5.** Dietary characteristics of the analysis sample (*n* 783) and by each time point

|  | Overall | |  | CDAH-1 | |  | CDAH-2* | |  | CDAH-3 | |
| --- | --- | --- | --- | --- | --- | --- | --- | --- | --- | --- | --- |
| Characteristic | Mean | (SD) |  | Mean | (SD) |  | Mean | (SD) |  | Mean | (SD) |
| Total energy intake (MJ/day) | 9·6 | (2·6) |  | 9·9 | (3·2) |  | 9·6 | (3·0) |  | 9·2 | (3·1) |
| Alcohol intake (g/day) | 17·2 | (18·8) |  | 17·4 | (23·3) |  | 15·2 | (19·7) |  | 17·9 | (25·2) |
| Dietary Guideline Index score | 41·5 | (8·4) |  | 41·6 | (9·8) |  | 42·7 | (9·6) |  | 41·1 | (9·9) |
| Red and processed meat† | 1·8 | (0·9) |  | 1·8 | (1·0) |  | 1·8 | (1·1) |  | 1·7 | (1·1) |
| Red meat | 1·3 | (0·7) |  | 1·4 | (0·8) |  | 1·4 | (0·8) |  | 1·3 | (0·9) |
| Processed meat | 0·4 | (0·3) |  | 0·5 | (0·4) |  | 0·4 | (0·4) |  | 0·4 | (0·4) |
| Plant-based alternatives† | 2·6 | (1·6) |  | 2·3 | (1·9) |  | 2·9 | (2·2) |  | 2·7 | (2·1) |
| Legumes | 0·6 | (0·5) |  | 0·6 | (0·6) |  | 0·6 | (0·6) |  | 0·6 | (0·7) |
| Nuts and seeds | 0·5 | (0·5) |  | 0·3 | (0·5) |  | 0·5 | (0·7) |  | 0·7 | (0·9) |
| Whole grains | 1·5 | (1·1) |  | 1·4 | (1·5) |  | 1·8 | (1·7) |  | 1·4 | (1·5) |

CDAH, Childhood Determinants of Adult Health; SD, standard deviation.

*Of the participants in the analysis sample, almost two-thirds had also completed a food frequency questionnaire at CDAH-2: 496 had a Dietary Guideline Index score and 531 had an estimate for remaining dietary variables.

†The g/day intake was scaled to the standard serve sizes used in the 2013 Australian dietary guidelines.

**Supplemental Table S6.** The modelled replacement of 0·5 serve/day of red and processed meat with plant-based alternatives and the estimated effect on log-HOMA2 insulin sensitivity (*n* 783)*

| Primary analysis† | *β* | 95% CI | *p* |
| --- | --- | --- | --- |
| Replacing 0·5 serve/day of red meat with… |  |  |  |
| Plant-based alternatives | 0·05 | 0·02, 0·08 | 0·001 |
| Legumes | 0·06 | 0·01, 0·10 | 0·010 |
| Nuts and seeds | 0·08 | 0·03, 0·12 | 0·001 |
| Whole grains | 0·06 | 0·02, 0·09 | 0·001 |
| Replacing 0·5 serve/day of processed meat with… |  |  |  |
| Plant-based alternatives | 0·04 | −0·03, 0·12 | 0·24 |
| Legumes | 0·07 | −0·01, 0·14 | 0·10 |
| Nuts and seeds | 0·08 | −0·001, 0·15 | 0·054 |
| Whole grains | 0·06 | −0·02, 0·13 | 0·12 |
| Replacing 0·5 serve/day of red and processed meat with… |  |  |  |
| Plant-based alternatives | 0·04 | 0·02, 0·07 | 0·001 |
| Legumes | 0·05 | 0·01, 0·09 | 0·016 |
| Nuts and seeds | 0·07 | 0·03, 0·11 | 0·001 |
| Whole grains | 0·05 | 0·02, 0·08 | <0·001 |

CI, confidence interval; HOMA2, homeostasis model assessment version 2·2.

*The change in log-transformed HOMA2 insulin sensitivity (percentage points) when simulating a 0.5 serve/day lower intake of red and processed meat with a concurrent 0.5 serve/day higher intake of plant-based alternatives (legumes, nuts and seeds, and whole grains).

†Adjusted for sex, age at blood draw, highest education, smoking status, physical activity, parental history of diabetes, use of hormonal contraceptive, use of blood pressure or cholesterol-lowering medication, energy intake, alcohol intake, and Dietary Guideline Index score.

**Supplemental Table S7.** Sensitivity analyses examining the modelled replacement of 1 serve/day of red and processed meat with plant-based alternatives and the estimated effect on log-HOMA2 insulin sensitivity*

| Sensitivity analyses relative to original analysis | *β* | 95% CI | *p* |
| --- | --- | --- | --- |
| Original analysis† | 0·10 | 0·04, 0·16 | 0·001 |
| Further adjusted for waist circumference (*n* 781) | 0·04 | −0·01, 0·09 | 0·12 |
| All-components model‡ | 0·06 | 0·00, 0·13 | 0·048 |
| Further adjusted for waist circumference (*n* 781) | 0·04 | −0·01, 0·09 | 0·15 |
| Exclusion of health conditions at CDAH-1 (*n* 674) | 0·09 | 0·02, 0·15 | 0·006 |
| Exclusion of infrequent and non-consumers (*n* 739)§ | 0·11 | 0·04, 0·17 | 0·002 |
| Inverse probability weighting procedure | 0·08 | 0·002, 0·15 | 0·043 |

CI, confidence interval; HOMA2, homeostasis model assessment version 2·2.

*The change in log-transformed HOMA2 insulin sensitivity (percentage points) when simulating a 1 serve/day lower intake of red meat with a concurrent 1 serve/day higher intake of plant-based alternatives (legumes, nuts and seeds, and whole grains). The sample size for analyses was 783 unless stated otherwise.

†Adjusted for sex, age at blood draw, highest education, smoking status, physical activity, parental history of diabetes, use of hormonal contraceptive, use of blood pressure or cholesterol-lowering medication, energy intake, alcohol intake, and Dietary Guideline Index score.

‡Adjusted for the same covariates in the original analysis (except for energy intake and Dietary Guideline Index score) in addition to all other energy-providing food groups (in g/day), including fruit, vegetables, tea and coffee, fruit and vegetable juices, salad dressings, refined grains, milk, yoghurt, cheese, eggs, seafood, poultry, sugar-sweetened beverages, artificially sweetened beverages, takeaway foods, mixed discretionary foods, and processed meat.

§Defined as < 0·3 serve/day of red meat or plant-based alternatives.

# References

1. Wilson JE, Blizzard L, Gall SL *et al.* (2019) An age- and sex-specific dietary guidelines index is a valid measure of diet quality in an Australian cohort during youth and adulthood. *Nutr Res* **65**, 43-53.

2. National Health and Medical Research Council, Australian Government Department of Health and Ageing, New Zealand Ministry of Health (2006) *Nutrient reference values for Australia and New Zealand*. Canberra, Australia: National Health and Medical Research Council.

3. Popkin BM, Armstrong LE, Bray GM *et al.* (2006) A new proposed guidance system for beverage consumption in the United States. *Am J Clin Nutr* **83**, 529-542.

4. Australian Bureau of Statistics (1986) *Census of Population and Housing: Socio-Economic Indexes for Areas (2033.0.55.001)*. Canberra, Australia.

5. Cole TJ, Bellizzi MC, Flegal KM *et al.* (2000) Establishing a standard definition for child overweight and obesity worldwide: international survey. *BMJ* **320**, 1240.
